# EASY-FIA: A Readably Usable Standalone Tool for High-Resolution Mass Spectrometry Metabolomics Data Pre-Processing

**DOI:** 10.3390/metabo13010013

**Published:** 2022-12-21

**Authors:** Aurelia Morabito, Giulia De Simone, Manuela Ferrario, Francesca Falcetta, Roberta Pastorelli, Laura Brunelli

**Affiliations:** 1 Laboratory of Mass Spectrometry, Istituto di Ricerche Farmacologiche Mario Negri IRCCS, 20156 Milan, Italy; 2 Department of Electronics, Information and Bioengineering, Politecnico di Milano, 20133 Milan, Italy; 3 Department of Biotechnologies and Biosciences, Università degli Studi Milano Bicocca, 20126 Milan, Italy; 4 Unit of Biophysics, Istituto di Ricerche Farmacologiche Mario Negri IRCCS, 20156 Milan, Italy

**Keywords:** Flow Injection Analysis-High Resolution Mass Spectrometry, FIA-HRMS data pre-processing, *m/z* alignment and annotation, metabolomics

## Abstract

Flow injection analysis coupled with high-resolution mass spectrometry (FIA-HRMS) is a fair trade-off between resolution and speed. However, free software available for data pre-processing is few, web-based, and often requires advanced user specialization. These tools rarely embedded blank and noise evaluation strategies, and direct feature annotation. We developed EASY-FIA, a free standalone application that can be employed for FIA-HRMS metabolomic data pre-processing by users with no bioinformatics/programming skills. We validated the tool′s performance and applicability in two clinical metabolomics case studies. The main functions of our application are blank subtraction, alignment of the metabolites, and direct feature annotation by means of the Human Metabolome Database (HMDB) using a minimum number of mass spectrometry parameters. In a scenario where FIA-HRMS is increasingly recognized as a reliable strategy for fast metabolomics analysis, EASY-FIA could become a standardized and feasible tool easily usable by all scientists dealing with MS-based metabolomics. EASY-FIA was implemented in MATLAB with the App Designer tool and it is freely available for download.

## 1. Introduction

Metabolism is at the cornerstone of all cellular functions, and it is deregulated in different and complex diseases. This emphasizes the importance of a comprehensive understanding of metabolic regulation at the whole-organism level [1]. Metabolomics has also been described as the “real-world endpoint” of omics research [2] and is closely linked to the phenotype of an organism [3]. Metabolic phenotypes are the result of the interplay between the genome and the environment, so metabolomics represents the organism’s response to perturbations due to either internal or external factors [4,5].

Untargeted metabolomics has been widely used as an unbiased strategy for the whole profiling of small molecules in biological systems, either to point out potential signatures and biomarkers of pathologies or to better understand their underlying mechanisms and progression. Mass spectrometry-based untargeted metabolomics is widely used to identify metabolic alterations associated with human disease [6,7,8,9,10,11,12,13,14]. Beyond that, untargeted metabolomics has been successfully applied in forensic, environmental, food, and agricultural studies. For instance, metabolomics could possibly be employed to determine putative biomarkers of drug consumption, pollution control, soil improvement and environmental monitoring by studying the metabolism of microorganisms [15]. Other fields which are exploiting metabolomics are precision nutrition, with the aim of assessing individual responses and profiles of dietary patterns in humans, and food analysis, from risk and safety assessment to quality evaluation [16]. Finally, metabolomics is increasingly employed in combination with other omics for the elucidation of various crop functions and responses to stress [17]. 

Liquid Chromatography coupled with Mass Spectrometry (LC-MS) is one of the most widely used techniques used in untargeted metabolomics due to its good selectivity and sensitivity allowing the detection of thousands of metabolites in a single analysis [18]. Direct injection of the sample into a carrier stream, without any chromatographic system (Flow Injection Analysis-High Resolution Mass Spectrometry, FIA-HRMS) has found even wider application in untargeted metabolomics as it offers similar advantages as LC-MS in terms of sensitivity, precision and accuracy, but greater method simplicity and fast analysis time [19]. FIA-HRMS has thus become a technique for untargeted metabolomics applications in clinical studies, providing analysis of many samples and fast screening time, as is the case for critically ill patients where patient stratification is time-sensitive [20]. FIA-HRMS has been also used in archaeological samples [21], to discriminate between different kinds of cannabinoids present in cannabis samples [22], or to characterize eventual food adulterations [23].

Metabolomics investigations usually include the collection and pre-processing of the MS raw data, statistical analysis to detect features of interest, and feature identification or annotation to cast light on their chemical and biological functions [24]. Several standardized data pre-processing protocols are available for untargeted LC-MS [25,26,27,28,29,30] and Nuclear Magnetic Resonance [31]; however, algorithms for FIA-HRMS data pre-processing are few and web-based. This is probably due because each type of mass spectrometer produces data in a proprietary format; therefore, data pre-processing depends on the software distributed by the vendor [32]. Furthermore, only a limited number of free tools merge data pre-processing with feature imputation using databases (e.g., Human Metabolome Database—(HMDB), Kyoto Encyclopedia of Genes and Genomes—(KEGG), and LIPID MAPS), in a single pipeline useful for all type of users, beginners included [33]. For instance, FIA-HRMS pre-processing might be aided by the proFIA software, which is available either as an R package on the Bioconductor repository [34] or as a tool of Workflow4Metabolomics online infrastructure in the Galaxy environment [24]. proFIA implements a strategy to pre-process FIA-HRMS raw data and generates the peak table; however, it does not provide an embedded method for blank subtraction and direct feature annotation. Our present work aims at providing the applicability and performance of easy-to-use standalone software for FIA-HRMS metabolomics data pre-processing.

### EASY-FIA Approach

**Figure 1:**
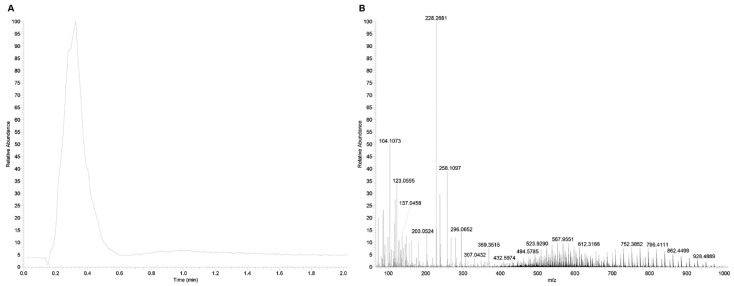
FIA-HRMS spectrum acquisition. (**A**): FIA-HRMS representative recorded spectrum; (**B**): *m/z* feature distribution obtained using the FIA-HRMS approach.

FIA-HRMS experiment is characterized by the direct injection of sequential samples directly into the mass spectrometer source without prior chromatographic separation. FIA-HRMS via modern high-resolution mass spectrometers such as Orbitrap and time-of-flight (ToF) was shown to enable the determination of hundreds to thousands of *m/z* features in biological samples allowing the efficient discrimination between isobaric compounds and facilitating the determination of molecular formulas, providing key technology for high-throughput metabolomics analysis [35]. Each FIA-HRMS data file consists of successive acquisitions of mass spectra (*m/z* value) within a defined time window (typically one-two minutes) and has a classical two-dimensional structure (*m/z* and intensity) as shown in Figure 1.

A fast analysis is pivotal to ensure that large sample cohorts are analyzed within a reasonable time and with optimal allocation of experimental resources. Metabolomics clinical study size can quickly scale to several or tens of thousands of individual samples to fulfill the demand imposed by robust experimental design. Large-scale phenotyping analysis can hardly be performed in a short time when the chromatographic step is included, because of the daily limits of the acquired samples. 

Here, we set out to establish a platform for routine high-throughput and high-mass-accuracy metabolomics data pre-processing, which combines metabolic feature extraction, blank subtraction, feature alignment, and annotation to generate a single table of metabolic features by their intensities. Together with a robust and reliable feature alignment to avoid false positives, first-pass automatic feature annotation is fundamental to speed up metabolomics data elaboration. EASY-FIA implements a non-chromatographic-based *m/z* annotation using an in-house HMBD-derived database to annotate single or multiple accurate *m/z* features by a customizable adduct annotation list of [M+H]^+^/[M−H]^−^ candidates. First-round identification by full-scan mass spectra information needs to be completed with tandem (MS/MS) mass spectral data for metabolite identification.

In the following sections, we describe EASY-FIA workflow algorithms (Materials and Methods) and the performance of our tool using two clinical metabolomics datasets (Results). Furthermore, we examined the evaluation of the applicability of blank subtraction and the unbiased mathematical definition of a peak intensity threshold to discard spurious intensities. 

We expect EASY-FIA to be highly useful for high-throughput clinical metabolomics applications (e.g., population-level disease screening and omics data integration strategies).

## 2. Materials and Methods

### 2.1. EASY-FIA Pre-Processing Workflow

EASY-FIA software for FIA-HRMS metabolomics pre-processing was implemented in MATLAB (version R2021a) environment. If the user does not have a currently available license of MATLAB software, a MATLAB runtime can be downloaded free of charge from the MATLAB website; it is a standalone set of shared libraries that enables the execution of compiled MATLAB components such the one proposed in this work. The required MATLAB Runtime is the release R2021a (Version 9.10). Finally, EASY-FIA can be freely downloaded either from GitHub at https://github.com/AMrbt20/EASY-FIA/ (accessed on 15 December 2022) under a GNU GPL v3.0 license, or at https://www.marionegri.it/centro-di-ricerca-spettrometria-di-massa-per-la-salute-e-ambiente (accessed on 15 December 2022) by following the provided instructions. The user-friendly Graphical Computer Interface (Supplementary Figure S1) aids to set a minimum number of mass spectrometry parameters for data processing (*m/z* tolerance interval, adducts, and intensity/sample cut-off values). Additional documentation and instructions are available on GitHub. 

**Figure 2:**
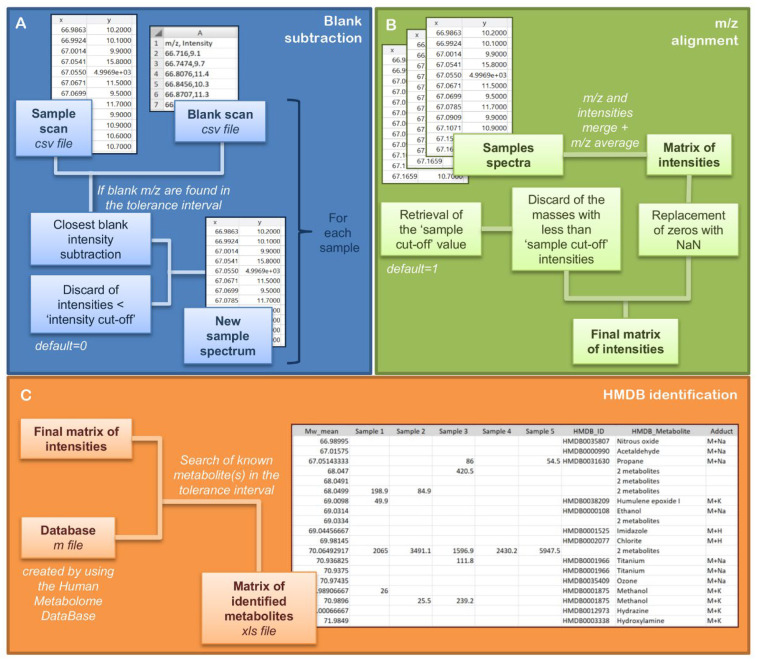
Schematic representation of EASY-FIA pipeline. Each key step of the algorithm is represented by a colored box. (**A**) Blank subtraction for noise filtering; (**B**) *m/z* alignment section for sample feature alignment; (**C**) HMDB identification for a first-round identification of the aligned *m/z*.

The EASY-FIA pipeline consists of three steps: blank subtraction, *m/z* feature alignment and feature annotation using the in-house HMDB database (Figure 2).

#### 2.1.1. Blank Subtraction

The *m/z* profile of a sample can be contaminated by compounds present in the carrier solvent. As the carrier flow is constant throughout the FIA-HRMS experiment, as demonstrated in the result section, the contribution of the solvent compounds to the intensity of the analyte can be managed easily. The function ‘ALIGNMENT’ takes as input the centroided *m/z* list exported into .csv format from FIA-HRMS raw files. Each sample must have its corresponding blank in the sample acquisition list. The blank is the solvent carried and the extraction solvent used in the experiment (Figure 2A). The algorithm searches each sample-blank pair for any *m/z* correspondence within a tolerance interval: a cycle is employed in order to loop over each *m/z* of the sample and search for any *m/z* correspondence in the blank within the defined tolerance interval. The tolerance value (t) must be inserted in the *Delta mass (ppm)* field of the GUI before starting processing. It is recommended to set this value equal to the external calibration range boundary of the high-resolution mass spectrometer used for the FIA-HRMS analysis. When an *m/z* of the sample matches its blank, the intensity of the blank *m/z* is subtracted from the sample one. If multiple matches are found within the tolerance interval, the sample *m/z* and all the relative blank *m/z* values will be written in a secondary table in which the distance in ppm will be calculated for each match, and only the closest blank *m/z* will be selected for blank subtraction. EASY-FIA also gives the user the possibility to discard the *m/z* whose intensities are under an arbitrary value in all samples. This cut-off has to be specified by the user in the *Intensity cut-off* field (Figure 2A).

#### 2.1.2. Alignment of m/z

Alignment starts from the first *m/z* of the first sample, and the algorithm searches each *m/z* in all the other *m/z* lists within the tolerance interval t using a while loop. When an *m/z* match is found within the t interval, the matching *m/z* is progressively averaged, and their mean is saved in an accessory vector. Then, the relative intensities of each matched *m/z* are reported sequentially in a single matrix, where the vector of averaged masses appears in the first column. The final matrix of intensities contains an identification number for each *m/z* to aid eventual subsequent analysis (Figure 2B).

Before starting the processing, the user can flag the *Sort matrix by adducts* option, available for the positive ionization mode. This function rearranges the matrix of intensities by sorting and grouping each M+H with its theoretical sodium and potassium adducts (M+Na, M+K). First, the algorithm subtracts the theoretical value of hydrogen from all *m/z* values in the matrix of intensities. Then, the algorithm adds the theoretical masses of sodium and potassium ions to each of the generated monoisotopic masses. The theoretical adducts are then searched in the experimental *m/z* in the matrix of intensity. When an *m/z* match is found within the tolerance interval, the row is moved under the corresponding M+H value. 

A matrix cleaning procedure automatically runs at the end of the alignment to replace the zeros in the matrix of intensities with NaN (not a number), to manage the absence of intensity values for specific *m/z.* The GUI also implements the *Sample cut-off* option, which removes the features whose intensity is not detected in a number of samples defined by the user (Figure 2B). The EASY-FIA default threshold is 1 (it removes *m/z* detected in just one sample), but the user can change this number in the *Sample cut-off* field of the GUI. In the end, the matrix of intensities is saved in the same folder in both MATLAB (.mat) and Excel file formats with the suffix _alignment_data_sorted or alignment_data according to the option selected.

#### 2.1.3. Human Metabolome Database (HMDB) Annotation

EASY-FIA automatically identifies the metabolic features (*m/z* value) by comparing each experimental *m/z* with the theoretical one present in the Human Metabolome Database (HMDB). This step can be run either independently or immediately after the alignment.

Two in-house databases were generated for annotation, one for positive and one for negative ionization mode; they are available as MATLAB files in the GitHub repository as HMDB_POS.mat and HMDB_NEG.mat, respectively. The HMDB in-house databases were created from the HMDB repository by downloading the XML file (All Metabolites, version 23 October 2021) and retrieving the identification code, metabolite name and monoisotopic weight for each metabolite. The in-house databases were obtained by adding the theoretical weights of hydrogen to the monoisotopic weight of the retrieved metabolites, sodium and potassium ions, for the positive acquisition mode, and subtracting the theoretical weight of hydrogen for the negative acquisition mode. EASY-FIA requires the user to load first the Excel file containing the matrix of intensities produced in the alignment section, then the HMDB mat file according to the ionization mode of the data under investigation. The algorithm looks for every *m/z* present in the matrix of intensities in the HMDB in-house database, implementing a cycle and a find function to search each mass within the tolerance interval. For any *m/z* match, the HMDB identification code, the name and the adduct of the metabolite are inserted in the corresponding row of the matrix of intensities. When an *m/z* matches more than one metabolic feature, then the number of matches is reported (Figure 2C).

The output of the identification process is saved as an Excel file in the current folder with the suffix HMDB_ID and consists of the cleaned matrix of intensities where each identified mass is associated with the name of the corresponding metabolite or the number of related metabolites.

### 2.2. Case Studies

EASY-FIA was used for the sample alignment of two published clinical studies, both with the objective of determining metabolites that cast light on different pathological conditions.

Case Study 1 [36]. A longitudinal population-based study that investigated frailty syndrome in older subjects, and aims at identifying metabolic hallmarks of the frailty syndrome. A total of 130 plasma samples were analyzed by the LTQ-OrbitrapXL mass spectrometer (Thermo Fisher Scientific) equipped with an electrospray source operated in negative and positive modes. Briefly, metabolites were extracted by adding cold methanol (4:1, MeOH: plasma) to the plasma samples (20 µL); samples were incubated at −80 °C for 20 min and then centrifuged for 15 min at 14,000× *g*. The supernatant was collected, dried under nitrogen, and suspended in 25 µL of 0.1% formic acid. Each run of the instrument was carried out by injecting 8 µL of sample extract at a flow rate of 50 µL/min of mobile phase consisting of isopropanol/water (60:40, v/v) buffered with 5 nM ammonium at pH 9 for negative mode and methanol/water (60:40, v/v) with 0.1% formic acid at pH 3 for positive mode. The source temperature was set to 240 °C with 25 L/in drying gas and a nebulizer pressure of 35 psig. Reference masses for internal calibration were used in continuous infusion during the analysis (*m/z* 210.1285 for positive and *m/z* 212.0750 for negative ionization). Mass spectra were recorded from *m/z* 50 to 1000. The eight quality controls (QC) acquired during the FIA-HRMS analysis were used to test EASY-FIA reproducibility. 

Case Study 2 [20]. The longitudinal population-based investigation, aimed at verifying whether different responses to therapy in the acute phase of shock were associated with different plasma metabolic patterns. Forty-two (42) plasma samples were analyzed by the 6550 iFunnel Q-TOF mass spectrometer (Agilent) equipped with an electrospray source operated in negative and positive modes. Metabolites were extracted by adding four volumes of cold methanol to the plasma sample (10 µL); samples were vortexed and incubated at −20 °C for 1 h and then centrifuged 10 min at 14,000× *g*. The supernatant was collected, dried in a SpeedVac and resuspended in 50 µL of 0.1% formic acid. The flow rate of the instrument was set to 150 µL of mobile phase consisting of isopropanol/water (60:40, v/v) buffered with 5 nM ammonium at pH 9 for negative mode, and methanol/water (60:40, v/v) with 0.1% formic acid at pH 3 for the positive mode. The source temperature was set to 320 °C with 15 L/min drying gas and a nebulizer pressure of 35 psig. Reference masses for internal calibration were used in continuous infusion during the analysis (*m/z* 121.050873, 922.009798 for positive and *m/z* 11.9856, 1033.9881 for negative ionization). Mass spectra were recorded from *m/z* 50 to 1100. Case 2, quantitative data of the metabolic species analyzed both by untargeted metabolomics (FIA-HRMS) and by target metabolomics (AbsoluteIDQ 180 kit, Biocrates, Innsbruck, Austria) [37] were used to demonstrate the correlation between peak intensity and metabolite concentrations (microM).

### 2.3. Statistical Analysis

Spearman correlation analysis was performed to verify the correlation between peak intensities and concentration (GraphPad Prism 9.2.0) for those metabolites quantified by both metabolomics strategies (untargeted FIA-HRMS and targeted AbsoluteIDQ 180 kit Biocrates).

## 3. Results and Discussion

### 3.1. EASY-FIA Performance on FIA-HRMS Clinical Metabolomics Case Studies

The reliability and applicability of the EASY-FIA algorithm were tested on two published case studies of human plasma FIA-HRMS untargeted metabolomics profiling obtained using two different high-resolution mass spectrometers [20,36]. The raw data files acquired from the vendor software were easily converted to .csv files, obtaining the centroided *m/z* list of the samples and the relative blanks of the two case studies. The tolerance interval was set to 6 ppm for both positive and negative ionization modes for case study 1 (LTQ OrbitrapXL), and to 20 ppm for case study 2 (6550 iFunnel Q-TOF), taking into consideration the different instrumental accuracy; the intensity cut-off was set to 0 and the peaks cut-off to 1. EASY-FIA aligned the samples of both acquisition modes in the two studies. The intensity matrix in case study 1 contained a number of *m/z* equal to 248,000 and 251,557 with an average of, respectively, 12,936 and 15,094 *m/z* per sample for the positive and negative modes, respectively. The matrix of intensities in case study 2 had 38,363 and 74,569 *m/z*, with averages of, respectively, 18,115 and 37,196 *m/z* per sample for the positive and negative modes, respectively (Supplementary Tables S1–S5).

After alignment, EASY-FIA automatically annotated the *m/z* features by using the in-house databases and identified by HMDB 35,017 *m/z* (25,134 positive, 9883 negative) in case study 1 and 20,084 *m/z* (12,024 positive, 8060 negative) in case study 2 (Supplementary Tables S1–S5). 

Since EASY-FIA provides the first-round identification exclusively based on the *m/z*, users should confirm identities through MS/MS approaches. For the metabolites that were annotated unambiguously (*m/z* with a single HMDB metabolite), we observed that FIA-HRMS analysis acquire both nonlipid and lipid metabolites (nonlipid ~93%, lipid ~6.3%) even though with a high prevalence of nonlipid metabolic species.

To evaluate the quality of the EASY-FIA pre-processing workflow, we compared the number of signals detected with peak intensities in the highest quartile (75th) because such features are expected to be less influenced by instrumental background and accurately quantified among the eight QC replicates. Seventy-one percent (71%) of the features were present in all QC replicates, highlighting the reproducibility of EASY-FIA. 

**Figure 3:**
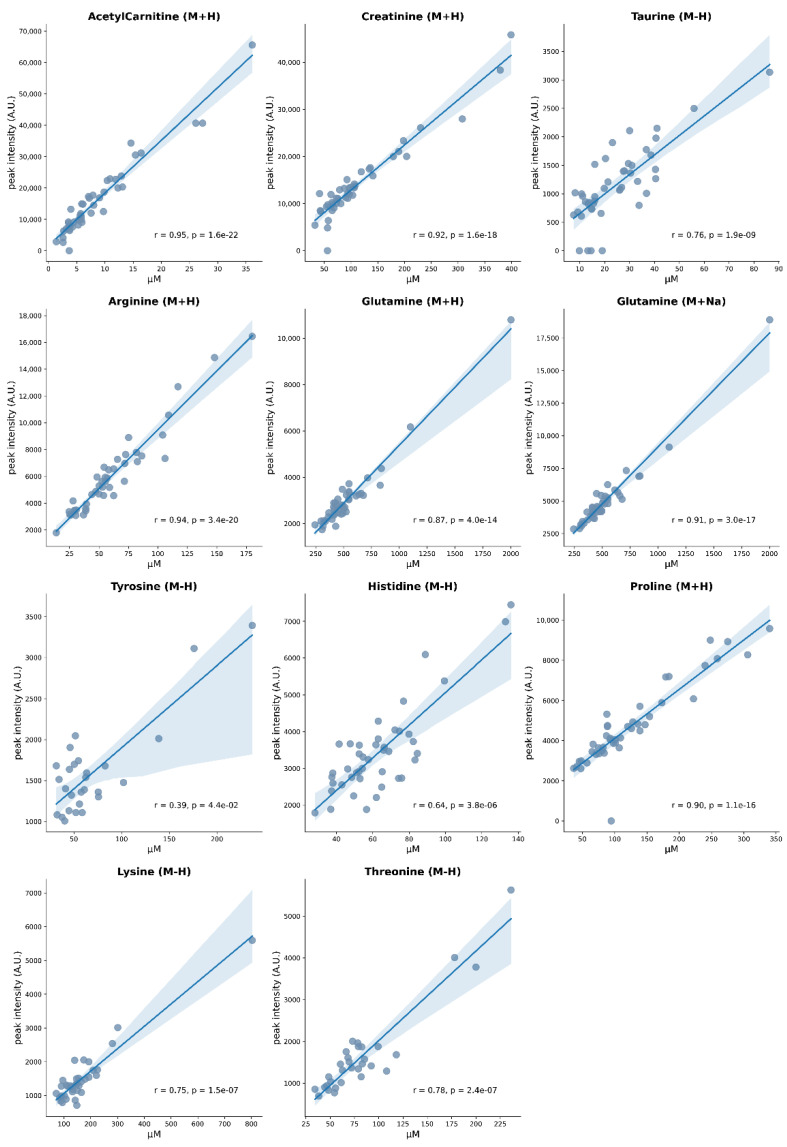
EASY-FIA validation. Spearman correlation between peak intensities and concentrations of metabolites quantified by untargeted FIA-HRMS and targeted AbsoluteIDQ 180 kit approaches. The blue line represents the regression line, blue dots refer to the samples and the shaded area represents the 95% confidence interval for that regression.

To further validate EASY-FIA performance, we verified the correlation between peak intensity and absolute concentration for those features quantified by both approaches (untargeted metabolomics by FIA-HRMS and targeted metabolomics by AbsoluteIDQ 180 kit Biocrates). Spearman correlation between peak intensities and concentrations for metabolic species belonging to amino acids (arginine, glutamine, tyrosine, histidine, proline, lysine, threonine), biogenic amines (taurine) and acylcarnitines (carnitine, acylcarnitine) showed a significant good correlation (r > 0.7 and *p*-value < 0.05) in both positive and negative ionization modes (Figure 3). As such, EASY FIA achieves performances of good linearity in terms of correlation between peak intensity and concentration. 

### 3.2. Unbiased Strategies for Limiting the Matrix Size of the Intensities

FIA-HRMS produces a huge matrix of intensities, with wide scattering due to a large number of missing values (*m/z* detected in a small number of samples or even in only one) in relation to the number of all the *m/z* detected (Table 1 and Supplementary Tables S1–S4). Thousands of *m/z* are generally detected in one single sample, and this poses a problem for statistical and data mining analyses. Moreover, high-dimensional matrices are subject to the so-called curse of dimensionality [38]: the performance of a machine learning model does not necessarily improve with the number of features, so they must be selected by removing noisy features and redundancies. 

Even though EASY-FIA is a tool for alignment and identification, and it aims at pre-processing data for further data mining applications, we tested some unbiased strategies for data cleaning and for dimensionality reduction by identifying and filtering non-relevant features. We searched for an objective cut-off intensity threshold to discard intensity values, instead of using an arbitrary threshold or hypothesis on noise characteristics.

### 3.3. Assessment of an Unbiased Strategy for Intensity Cut-Off to Remove Blank Spectra Noise

In the mass spectrometry-based metabolomics analysis, selecting the intensity cut-off to discard a portion of acquired *m/z* features is not a trivial issue. Indeed, a wrong value selection may have a detrimental impact on the subsequent data elaboration. To date, the criterion for selecting a cut-off intensity value is not univocal; in fact, several approaches can be seen in the scientific literature. For instance, Fuhrer et al. suggested filtering peaks of less than 500 ion counts in the summed spectrum [39]. Beuchel et al. implemented an algorithm for the removal of outliers based on the logarithmic transformation of non-zero measurements, and the threshold was set to be less than five timesthe standard deviation value (SD) [40,41]. Gatto et al. developed an R function called *removePeaks* to remove peaks with intensity below an arbitrary threshold [42]. However, the use of an arbitrary cut-off to discard *m/z* might be a biased strategy, considering the large value range of FIA-HRMS metabolomics matrices. 

We questioned the possibility of using the *m/z* spectrum of the blanks to establish an unbiased cut-off intensity value, which would be tailored to the intensity matrix of the investigated data. In case study 1, the intensity range goes from 13.6 arbitrary units (AU) to 1.08 × 10^7^ AU in positive mode, and from 14.6 to 4.11 × 10^6^ AU in negative mode. Case study 2 has an intensity range from 2.53 to 9.88 × 10^5^ AU for the positive mode and from 2.47 to 1.11 × 10^6^ AU for the negative mode. In case study 1, the blank had an intensity range between 14.60 and 7.53 × 10^5^ AU for positive mode and between 13.20 and 8.93 × 10^5^ AU for negative; in case study 2 the blank intensity ranged between 2.50 and 1.09 × 10^6^ AU for positive and 2.47 and 9.48 × 10^5^ for negative mode. 

**Figure 4:**
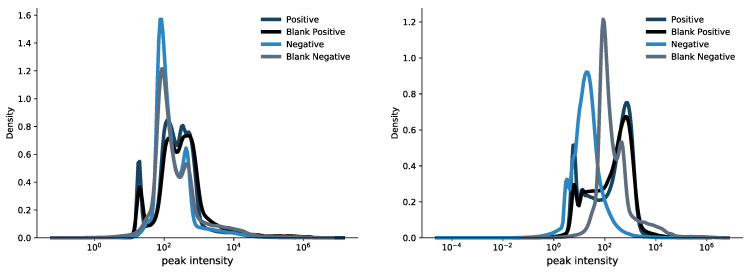
Distribution of peak intensity in the blank and sample spectra. The graphs show the peak intensity distribution density and thus the range of peak intensity in the blank and in the sample for case study 1 (**left panel**) and for case study 2 (**right panel**). *m/z* intensities are reported as arbitrary units and are displayed in logarithm scale.

We compared the distributions of peak intensities values between the blank and the samples in order to see if there were a putative cutoff value to be used as a threshold to separate them. As Figure 4 clearly shows the peak intensity distributions are completely overlapped and the threshold-based approach cannot be applied.

Note, that there was a low percentage of common *m/z* values (Supplementary Table S6) between blanks and samples, thus leading us to overlook the use of blank spectra for determining a cut-off threshold on peak intensities. Although we implemented the *intensity cut-off* functionality in EASY-FIA to allow the user to set a threshold under which intensities are discarded, we would like to emphasize that this biased strategy may affect the subsequent data elaboration, considering the high dynamic range of the acquired features.

### 3.4. Assessment of an Unbiased Strategy for m/z Reduction

**Figure 5:**
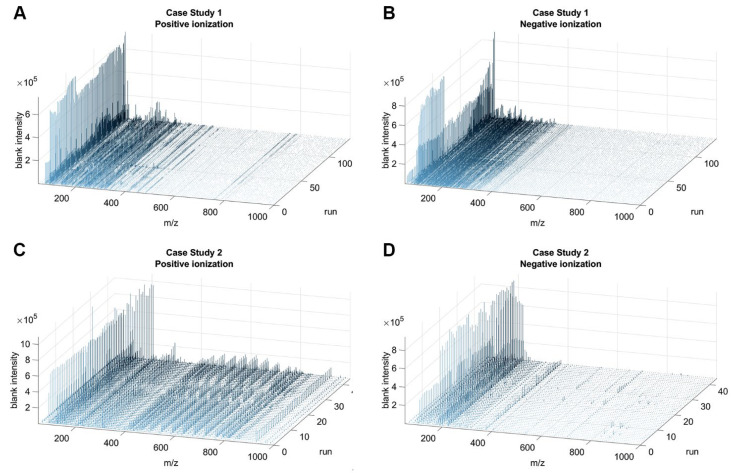
Blank spectra over runs. Three-dimensional plot of the *m/z* spectra of the blanks over the runs (y-axis) in the positive (**A**,**C**) and negative ionization (**B**,**D**) modes for each case study.

We evaluated the possibility of creating a mathematical model of the acquisition noise, in order to discard the *m/z* values associated with it. Several approaches have been proposed for the characterization of the acquisition noise [43], such as modeling baseline noise by using baseline functions (e.g., linear, logarithmic, exponential or piecewise [44]), the top-hat operator [45], or applying specific filters that remove the electronic noise based on the physics of the acquisition system [44]. We assume the acquisition noise to be contained in the spectrum of the blanks, so we evaluated the blank spectrum trend over the runs for positive (Figure 5A,C) and negative (Figure 5B,D) ionization modes in both case studies, seeking any temporal pattern. The blank spectra were characterized neither by a specific shape or trend that could be modeled by a mathematical function nor by any linear offset superimposed on the intensities due to the carry-over effect (Figure 5A–D). 

To further confirm these observations, we repeatedly picked 100 *m/z* per ionization mode in a random way, and we plotted their intensities through the runs in both case studies (Supplementary Figures S2 and S3). Since we had no evidence of a clear trend in the blank or a particular behavior depending on the run, EASY-FIA only implements the simple blank subtraction in order to painlessly remove the minimal contribution of the blank.

Overall, the impossibility of identifying an unbiased cut-off or a modellable noise for the FIA-HRMS analysis suggested setting the cut-off intensity to 0 (i.e., we took all the acquired *m/z*) and the sample cut-off to 1 (i.e., we discarded the *m/z* for which only one intensity was detected) for our analyses. 

Our strategy still enabled us to make a first features reduction: in case study 1, the *m/z* were reduced from 263,203 to 248,000 and from 263,061 to 251,557 in positive and negative mode, respectively, and in case study 2 from 53,314 to 38,363 and from 75,706 to 74,569, obtaining a matrix of intensities reduced by, respectively, the 7% and 17% for case study 1 and 2. If users wish to further reduce the number of *m/z* for subsequent data analysis, they may consider only the annotated features (i.e., case study 1: 35,017 annotated *m/z*, case study 2: 20,084 annotated *m/z*).

## 4. Conclusions

We develop EASY-FIA as a free, reliable, standalone tool for FIA-HRMS fast metabolomics data pre-processing; it is usable by users with no informatics/programming skills, and it only requires mass spectrometry parameters to be set up. Moreover, EASY-FIA automatically allows a non-chromatographic-based feature annotation according to the widely recognized Human Metabolome Database, thus providing rapid first-pass information about metabolic elements.

Performance on both data reproducibility (QC) and linearity (correlation with absolute metabolite concentrations) was validated, showing that EASY-FIA pre-processing achieves robust feature alignment. Furthermore, we demonstrated that EASY-FIA is intended for any kind of mass spectrometer data by applying our approach to two case studies of metabolomics clinical data obtained using two different high-resolution mass spectrometers. EASY-FIA implements an automatic blank subtraction to remove the blank’s minimal contribution since we assessed that neither an intensity cut-off value nor mathematical modeling of the noise worked as reliable methods for the efficient size reduction of the *m/z* intensity matrix. 

EASY-FIA code is fully embedded in an intuitive GUI and requires no programming skills. The GUI allows the user to customize a minimum number of alignment parameters related to the mass spectrometry tolerance interval, adducts, and intensity/sample cut-off values. The EASY-FIA metabolomics intensity matrix with huge numbers of metabolic features is then suitable for in-depth data analysis, including data mining, feature reduction, and importance ranking methods such as PLS-DA, SVM, RF, and mRMR [37] to select the features according to their importance in identifying the target class in the classification problem.

## Figures and Tables

**Table 1:** Missing values and Features’ intensity values in the two case study matrices. Number of missing values and intensity values detected in the intensity matrix for each case study, in accordance with the acquisition mode.

Case Study	Acquisition Mode	Missing Values	Intensity Values
1	Positive	32,290,973	1,685,027
1	Negative	32,762,205	1,952,661
2	Positive	1,168,071	519,901
2	Negative	2,343,890	937,146

## Data Availability

The data underlying this article are available in the Supplementary Materials and on GitHub at https://github.com/AMrbt20/EASY-FIA/.

## Supplementary Materials

The following supporting information can be downloaded at: https://www.mdpi.com/article/10.3390/metabo13010013/s1, Figure S1: Graphic User Interface of EASY-FIA; Figure S2: Case study 1-Multiple plots of 100 random *m/z* intensities (y-axis) through the runs (x-axis) for positive (A) and negative (B) ionization modes; Figure S3: Case study 2-Multiple plots of 100 random *m/z* intensities (y-axis) through the runs (x-axis) for positive (A) and negative (B) ionization modes.; Table S1: Matrix of intensities of case study 1 in positive ionization; Table S2: Matrix of intensities of case study 1 in negative ionization; Table S3: Matrix of intensities of case study 2 in positive ionization; Table S4: Matrix of intensities of case study 2 in negative ionization; Table S5: Summary of the number of *m/z* identified, not identified, and correctly aligned by EASY-FIA after discarding the *m/z* detected in a single sample; Table S6: Number of *m/z* acquired in the samples and blanks, in accordance with the acquisition mode. The table also reports the percentage of shared *m/z* between samples and blanks in both case studies.
