# Supplementary material for: EASY-FIA: A Readably Usable Standalone Tool for High-Resolution Mass Spectrometry Metabolomics Data Pre-Processing
Source: Metabolites. 2022 Dec 21;13(1):13. doi: 10.3390/metabo13010013 (PMC9861133; doi:10.3390/metabo13010013)
Supplement: Supplementary file 1 [file metabolites-13-00013-s001.zip › metabolites-2085105-supplementary.pdf]

EASY-FIA: A Readably Usable Standalone Tool for High-Resolution Mass Spectrometry Metabolomics Data Pre-Processing

Aurelia Morabito [1,2], Giulia De Simone [1,3], Manuela Ferrario [2], Francesca Falcetta [4], Roberta Pastorelli [1] and Laura Brunelli [1,*]

1 Laboratory of Mass Spectrometry, Istituto di Ricerche Farmacologiche Mario Negri IRCCS, Milan, Italy

2 Department of Electronics, Information and Bioengineering, Politecnico di Milano, Milan, Italy.

3 Department of Biotechnologies and Biosciences, Università degli Studi Milano Bicocca, 20126 Milan, It-aly

4 Unit of Biophysics, Istituto di Ricerche Farmacologiche Mario Negri IRCCS, Milan, Italy

Supplementary materials

Supplementary figure 1: Graphic User Interface of EASY-FIA

Supplementary figure 2: Case study 1-Multiple plots of 100 random *m/z* intensities (y axis) through the runs (x axis) for positive (A) and negative (B) ionization modes.

Supplementary figure 3: Case study 2-Multiple plots of 100 random *m/z* intensities (y axis) through the runs (x axis) for positive (A) and negative (B) ionization modes.

Supplementary Table 1: Matrix of intensities of case study 1 in positive ionization. https://github.com/AMrbt20/SupplementaryMaterials

Supplementary Table 2: Matrix of intensities of case study 1 in negative ionization.

https://github.com/AMrbt20/SupplementaryMaterials

Supplementary Table 3: Matrix of intensities of case study 2 in positive ionization.

https://github.com/AMrbt20/SupplementaryMaterials

Supplementary Table 4: Matrix of intensities of case study 2 in negative ionization.

https://github.com/AMrbt20/SupplementaryMaterials

Supplementary table 5: Summary of the number of m/z identified, not identified, and correctly aligned by EASY-FIA after discarding the m/z detected in a single sample.

Supplementary table 6: Number of *m/z* acquired in the samples and blanks, in accordance with the acquisition mode. Table also reports the percentage of shared *m/z* between samples and blanks in both case studies.

Supplementary Figure S1: Graphic User Interface of EASY-FIA.

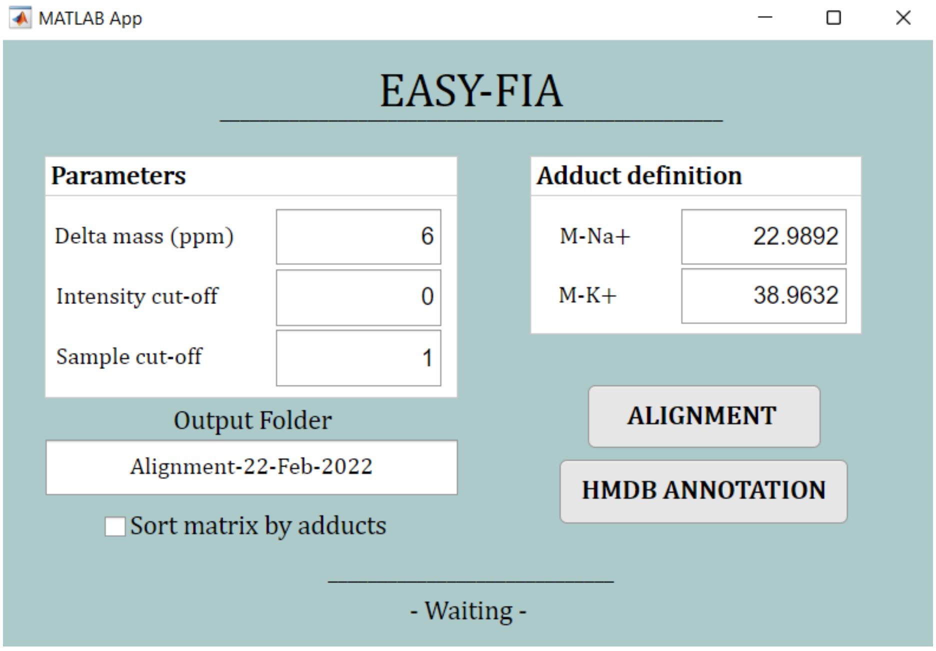

Supplementary Figure S2: Case study 1-Multiple plots of 100 random *m/z* intensities (y axis) through the runs (x axis) for positive (A) and negative (B) ionization modes.

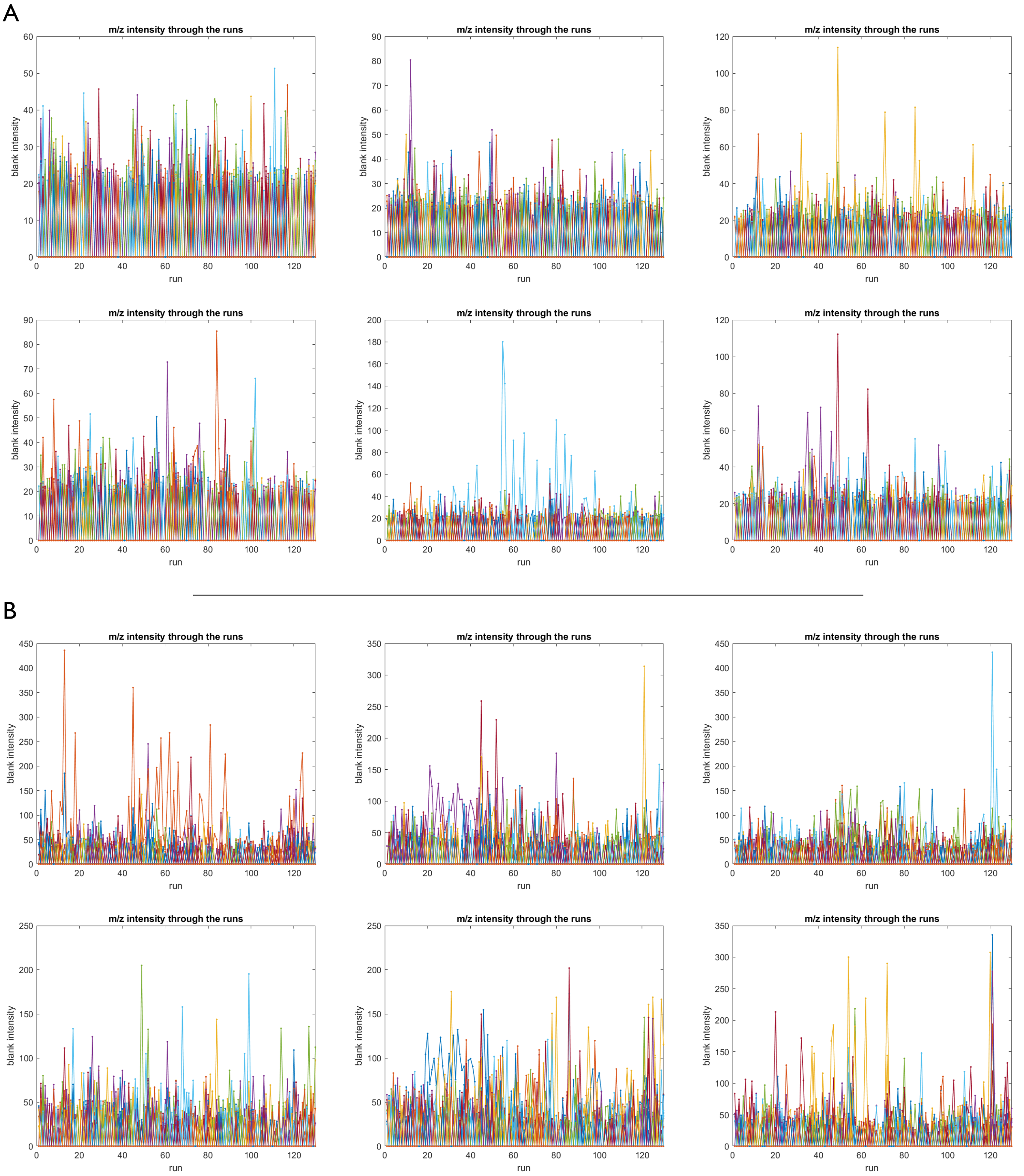

Supplementary Figure S3: Case study 2-Multiple plots of 100 random *m/z* intensities (y axis) through the runs (x axis) for positive (A) and negative (B) ionization modes.

A

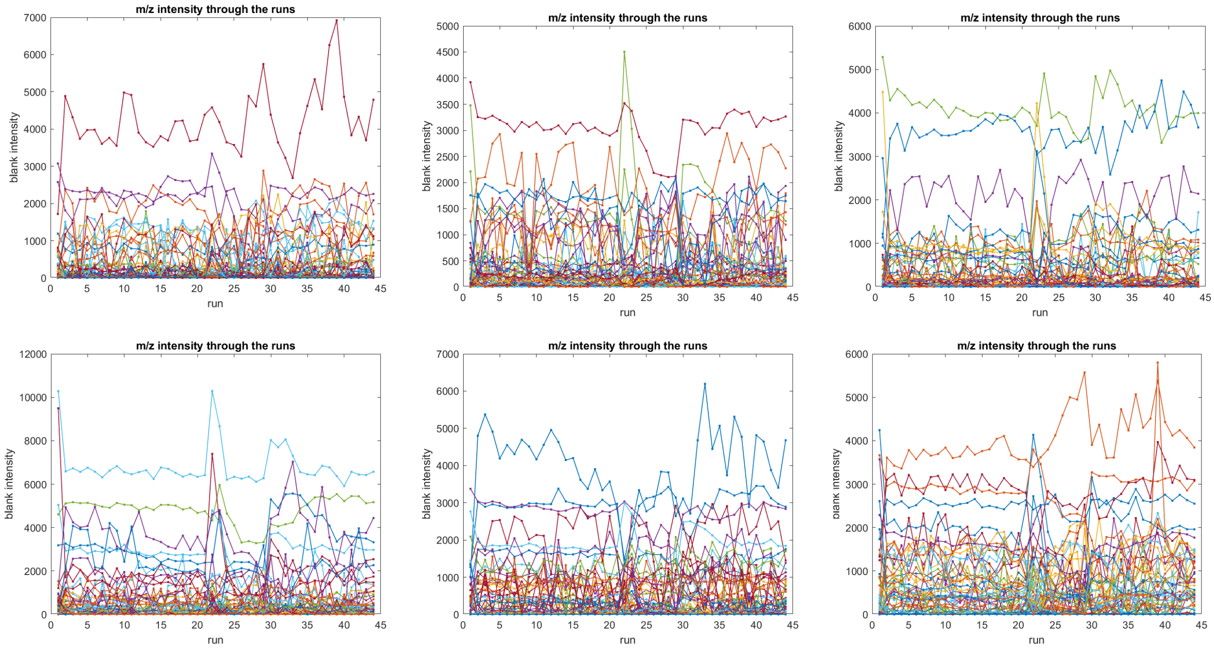

B

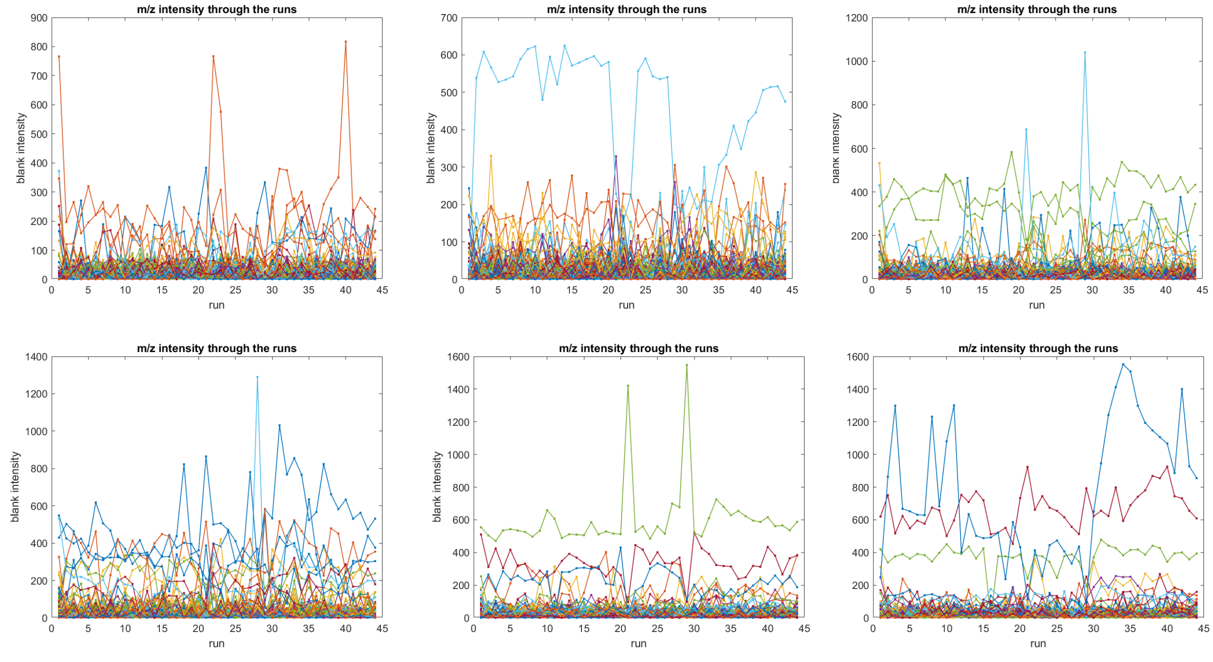

Supplementary Table S5: Summary of the number of *m/z* identified and not identified, correctly aligned by EASY-FIA after discarding the *m/z* detected in a single sample.

Case Study	Acquisition mode	Identified *m/z*	Not identified *m/z*	Tot. m/z	Tot. identified *m/z*
1	Positive	25.134	222.866	248.000	35.017
1	Negative	9.883	241.674	251.557	
2	Positive	12.024	26.339	38.363	20.084
2	Negative	8.060	66.509	74.569	

Supplementary Table S6: Number of *m/z* acquired in the samples and blanks, in accordance with the acquisition mode. Table also reports the percentage of shared *m/z* between samples and blanks in both case studies.

**Case Study**	**Acquisition mode**	**Sample *m/z***	**Blank *m/z***	**Sample/Blank *m/z* match (%)**
1	Positive	263.122	262.933	21,43
1	Negative	262.943	262.726	27,47
2	Positive	54.885	35.687	38,19
2	Negative	74.799	74.866	96,54
